# Healthy Aging and Compensation of Sentence Comprehension Auditory Deficits

**DOI:** 10.1155/2015/640657

**Published:** 2015-10-27

**Authors:** Marcela Lima Silagi, Camila Maia Rabelo, Eliane Schochat, Letícia Lessa Mansur

**Affiliations:** Department of Physical Therapy, Speech-Language Pathology and Occupational Therapy, School of Medicine, University of São Paulo, 05360-000 São Paulo, SP, Brazil

## Abstract

*Objectives*. To analyze the effect of aging on sentence auditory comprehension and to study the relationship between this language skill and cognitive functions (attention, working memory, and executive functions). *Methods*. A total of 90 healthy subjects were divided into three groups: adults (50–59 years), young-old (60–69 years), and old-old (70–80 years). Subjects were assessed using the Revised Token Test. The measures used for performance analysis were number of correct answers (accuracy) and execution time of commands on the different subtests. *Results*. Regarding accuracy, groups showed similar performance on the first blocks, but the young-old and old-old performed worse than adults on blocks 9 and 10. With respect to execution time, groups differed from block 2 (i.e., the groups differed for all blocks, except for block 1), with the worst performance observed in the old-old group, followed by that of the young-old group. Therefore, the elderly required more time to attain performance similar to that of adults, showing that time measurements are more sensitive for detecting the effects of age. Sentence comprehension ability is correlated with cognitive test performance, especially for global cognition and working memory tests. *Conclusions*. Healthy aging is characterized by the ability to compensate for difficulties in linguistic processing, which allows the elderly to maintain functional communication.

## 1. Introduction

It is generally agreed that aging causes language disorders in the elderly, but this issue has not received sufficient research attention. With advancing age, disparities in cognitive performance and language among individuals increase. Studies have shown that the aging process causes heterogeneous changes in language and have sought to explain the reasons for the decline observed in certain functions and the sparing of others [1, 2].

There is extensive literature about changes in oral production with aging. It is known that vocabulary begins to decline starting at 50 years of age, whereas phonetic-phonological skills remain largely intact until very old age [3, 4]. Regarding language comprehension, a smaller number of investigations seeking to establish the effects on auditory processing deficits are observed. Auditory comprehension of sentences, especially of complex phrases, is an aging-related complaint [5], but the underlying causes of this decline and why some elderly subjects do not develop this deficit remains unclear.

Hearing deficits, both central and peripheral, are important factors to consider in language comprehension in the elderly [6, 7]. Moreover, the neural network of language comprehension processing is intrinsically related to other cognitive functions networks, such as working memory, attention, and executive function [8–10].

One of the mechanisms that could explain cognitive-linguistic heterogeneity manifested in normal elderly is cognitive reserve [11]. Brain networks are associated with cognitive reserve in adults, and compensation mechanisms can be observed in older elderly [12], as a result of the plasticity of the central nervous system [13].

Studies involving neuroimaging show inter- [14] and intrahemispheric reorganizations [15] associated with compensation during the aging process. Compensation becomes evident when images are correlated with stability or superiority of cognitive performance on several tasks, including language tasks [16].

One of the most widely used tests for assessing auditory sentence comprehension is the Token Test (TT) [17], which was devised to evaluate auditory memory and syntactic comprehension. The TT explores the ability to retain a significant number of items grouped into sections.

The TT has been the subject of several studies and is available in short versions [18–20], which have proven to be as effective as long versions for the detection of pathological conditions such as aphasia [18, 21–23] and neurodegenerative diseases [24–26].

Studies on the effects of sociodemographic variables in subjects without brain injury show a strong educational effect on performance in the TT, but the effect of aging is controversial, depending on the version of the test employed, the scoring system used, and the age range studied, with decline being most evident beyond 65 years of age [21, 27–30].

The study by Kim et al. [31] is notable for having used a response time measurement (measured from the end of the command until the initial touch of the piece, at natural speed) to verify the correlations between age and test performance, stating that these time measurements can be more sensitive for detecting the effects of aging.

Against this background, the main objective of this study was to compare the performance of adult, young-old, and old-old subjects in auditory sentence comprehension using the Revised Token Test (RTT) [22]. To study the effect of aging on this linguistic task, we used the number of correct answers (accuracy) and execution time of commands in the different subtests of the test.

An additional aim was to study the correlation of sentence comprehension ability with cognitive functions. The cognitive tests used for correlation with RTT were the Mini-Mental State Examination (MMSE) [32, 33] (global cognitive screening), semantic verbal fluency (FVS) [34] (semantic memory and executive functions), direct digit span [35] (attention and information storage capacity), and reverse digit span [35] (working memory and mind control for the operationalization of information).

The present study made differential use of the time measure and the execution time measure, because it is assumed to be more sensitive for detecting performance differences between age groups and allows researchers to observe the use of cognitive strategies for the implementation of full commands (manipulation of the tokens) and not only the timing between the giving of the command and the initial touch, for measuring the reaction time and response time. To the best of our knowledge, no studies using this form of time measurement for evaluating performance on the TT are available.

This study began with the hypothesis that auditory comprehension is a vulnerable skill in aging, but a subgroup of the healthy population retains performance similar to that of younger people. Elderly who maintain high performance do so through compensation mechanisms, which are related to the recruitment of cognitive functions that sustain language (e.g., attention, executive function, and working memory).

## 2. Materials and Methods

### 2.1. Ethics

This study is part of the larger project “Aging Maintaining Functions: elderly in the 2020s” run by the Department of Physical Therapy, Speech Therapy and Occupational Therapy, School of Medicine, University of São Paulo, Brazil. The study was supported by the National Council of Scientific and Technological Development (CNPq; process number 557887/2009-7) and was approved by the Research Ethics Committee of the University Hospital of the Medical School, University of São Paulo (registration CEP-HU/USP: 1005/10; SISNEP CAAE: 0034.0.198.000-10). After receiving complete information about the procedures, participants signed the consent form.

### 2.2. Participants

The study sample comprised 90 healthy subjects of both genders, aged 50 to 80 years, with over 5 years of formal education who were native Brazilian Portuguese speakers without cognitive complaints, with functional hearing and vision, with no motor deficits. Participants were equally divided into three groups according to age: Group 1, adults (50–59 years); Group 2, young-old (60–69 years); and Group 3, old-old (70–80 years).

#### 2.2.1. Inclusion Criteria

To be eligible for study enrollment, participants had to meet the inclusion criteria for studies in neuropsychology described in the Mayo Older American Normative Studies (MOANS) [36]. These criteria primarily include an absence of cognitive complaints or psychiatric/neurologic disorders, no recent use of psychoactive drugs, and no alcohol dependence.

The subjects were submitted to cognitive tests and asked about communication functionality in everyday life. Inclusion in the study was conditional on obtaining scores consistent with normative values for the Brazilian population on the following tests:

- Mini-Mental State Examination (MMSE) [32, 33]: it is used for cognitive screening, with a minimum score of 25 points for individuals with 1–4 years of education, 26 points for 5–8 years, 28 points for over 8 years, and 29 points for over 11 years of education.
- Adapted Cognitive Change Questionnaire (QMC) [37]: it includes questions about changes in performance of complex activities of daily living. The adopted cut-off score was 2 points.
- Geriatric Depression Scale-15 [38, 39]: it is used to detect depressive symptoms that could impact cognitive performance. The adopted cut-off score was 5 points.
- Functional Assessment of Communication Skills for Adults (ASHA-FACS) [40, 41]: the social communications domain was used for scoring. The test consists of 21 questions on the implementation of tasks independently, with different levels of assistance (minimum, minimum-moderate, moderate-maximum, and maximum) or impossibility of fulfillment. The final score is obtained by calculating the arithmetic mean of the scores attained on each question, with a maximum of 7 points.

All groups performed audiometric exams, with hearing thresholds of up to 40 dB horizontal line (HL) (at 500, 1000, and 2000 Hz), a symmetrical hearing configuration, and the presence of a V wave evoked with a click stimulus in the auditory brainstem response (ABR) (difference of up to 0.2 ms between the ears).

The subjects also underwent evaluations with an ophthalmologist and physiotherapist for exclusion of visual and motor abnormalities that may have compromised the tests run.

#### 2.2.2. Exclusion Criteria

Subjects with scores below the cut-off score on cognitive tests, depressive symptoms, and other psychiatric or neurological disorders were excluded. Subjects who failed the auditory, visual, and motor tests were also removed from the sample.

### 2.3. Procedures

#### 2.3.1. Revised Token Test (RTT)

To compare the performance of adults, young-old, and old-old subjects auditory sentence comprehension, we used the Brazilian Portuguese version of the RTT. The test consists of 50 commands grouped into ten sections. The task entailed manipulating tokens of different shapes, sizes, and colors: 20 tokens of five different colors (blue, red, green, white, and black), two formats (squares and circles), and two sizes (small and large).

Semantic content and cultural factors are minimized because the requested information is reduced to the size, shape, and color of the tokens.

However, there are a progressive number of requests, and extent of information, in order to recruit working memory. Subtests 1 to 4 have simple and composite imperative statements that assess understanding of color, size, and shape. Subtests 5 to 8 require comprehension of prepositions related to visual-spatial content in the handling of one part (active part) over another (inactive part). In the last two subtests (9, 10), there is an increase in both information and linguistic complexity by introducing prepositional phrases, adverbial clauses, and compound sentences combined in heterogeneous commands on many dimensions, as shown in Table 1. Adequate performance of commands requires the cognitive support of attention, working memory, executive function skills, and language processing at different levels [22].

The test was applied individually in a quiet environment, as recommended in the original manual. Pretest instructions were given to familiarize the subjects with the concepts of color, shape, and size. All commands were given aloud by a speech therapist experienced in language assessment who was a native speaker of Brazilian Portuguese.

The commands were given at a normal speech rate for Brazilians living in São Paulo [42] and at an intensity of 60–70 dB sound pressure level (SPL) or approximately 50 dB hearing level (HL). Regarding prosody, the presentation of each unit in each command had no special inflection or pauses between units; that is, the prosodic features (speed, fluency, emphasis, intonation, and articulation) were constant across commands.

#### 2.3.2. Cognitive Evaluation

To study the relationship between cognitive functions and sentence comprehension ability, the score and the total execution time in the RTT were correlated with the following tests:

- Mini-Mental State Examination (MMSE) [32, 33]: it is used for cognitive screening. The examination presents temporal and spatial orientation, immediate memory, attention and calculation, delayed recall, language (reading, writing, naming, and repetition) subtests, and design copy for assessing visuospatial skills.
- Semantic verbal fluency (FVS), animal category [34]: it evaluates semantic memory and executive functions. Subjects were instructed to list as many animals as they could in one minute.
- Digit span in direct and reverse order [35]: the subjects should repeat increasing sequences of numbers in direct and reverse order. The direct sequence evaluates attention and information storage capacity, whereas the reverse sequence assesses working memory and mind control for the operationalization of information.

### 2.4. Data Analysis

Performance on the RTT was analyzed with respect to number of correct answers (accuracy) and execution time of commands. For number of correct answers, one point was awarded for each correct answer. The analysis considered the sum of correct answers under the total score and on each subtest. The analysis of execution time (time difference between end of command and full completion of action: touching or manipulating the pieces) was timed and measured in seconds. The analysis considered the sum of the times taken on each block and the total time.

For descriptive analysis, means and standard deviations of all demographic variables and performance on cognitive tests and on the RTT for the three age groups were calculated. Comparison of means for continuous data was performed using one-way ANOVA, given the Gaussian distribution of the data. When the difference between groups was statistically significant, a post hoc (Bonferroni) test was applied for pairwise comparison.

The distribution of subgroups according to gender was compared by Pearson's chi-square test. Pearson's coefficient was calculated to determine the association between performance on the RTT and cognitive performance. The same test was also used to verify the correlation between number of correct answers and execution time on the RTT.

A 5% level of statistical significance was adopted for all analyses. Analyses were performed using the statistical software program BIOESTAT 5.0 [43].

## 3. Results

### 3.1. Demographic, Cognitive, and Communicative Functionality Characteristics

The demographic characteristics of the sample and performance on the cognitive tests are presented in Table 2. All groups differed with respect to age, but there was no statistically significant difference for the other variables, including education, which shows that the groups were well matched and homogeneous, allowing for the observation of aging effects.

### 3.2. Age Effect on RTT: Number of Correct Answers (Accuracy) and Execution Time

The performance of the groups, as measured by the number of correct answers on each subtest and total RTT, is presented in Table 3. All groups showed similar performance on most subtests (1 to 8), but adults differed from young and old-old on the last two subtests (9 and 10).

Table 4 shows the performance of the groups regarding execution time for each subtest and for the total test. The groups showed similar performance on subtest 1. The old-old group required a significantly longer time to perform the RTT test than did the young-old and adult groups. The old-old subjects differed from adults on all subtests (except for subtest 1) and from the young-old on subtests 2 and 6. The young-old group showed a longer execution time than adults on subtests 5, 8, and 9 and a longer total time.

**Figure 1:**
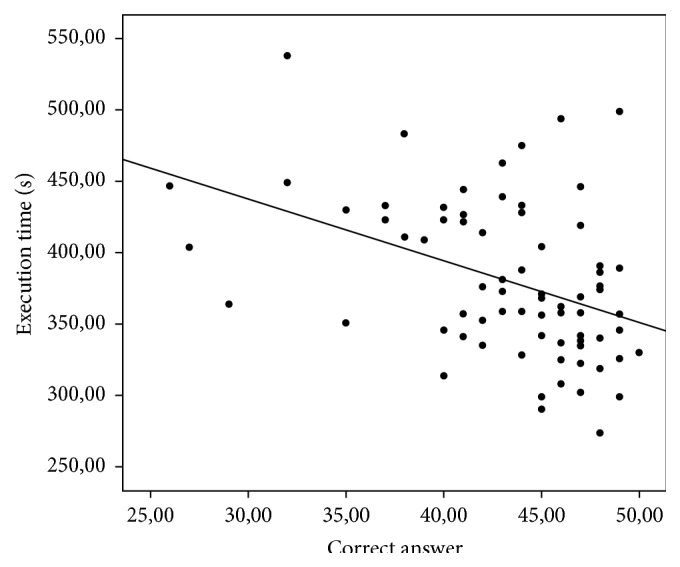
Correlation between number of correct answers (accuracy) and execution time on each RTT subtests.

Although the number of correct answers did not differentiate the groups with respect to total score, Pearson's correlation coefficient revealed that the variable number of correct answers had a statistically significant negative correlation with execution time (*p* < 0.001); that is, the longer the execution time, the lower the number of correct answers on the test, as shown in Figure 1.

### 3.3. Correlations between Performance on RTT and Cognitive Tests

Although the three groups exhibited no differences in education, in MMSE, or on the communication functionality questionnaire, some variables were correlated with group performance as a whole on the RTT, as shown in Table 5. There was a significant correlation between total number of correct answers on the RTT and MMSE score, digit span in direct and reverse order, and education (positive correlations).

## 4. Discussion

The results of this study show an age effect on auditory sentence comprehension, only for more complex commands. For number of correct answers, the groups exhibited similar performance, differing only on the last two subtests (adults performed better than the young-old and the old-old). However, the measurement of execution time differentiated the groups with respect to subtest 2. In general, the time required to complete the task increased with age. Additionally, there was a correlation between the number of total correct answers on the RTT, education, and performance on the cognitive tests: MMSE (global cognitive measures), direct digit span (attention), and reverse digit span (working memory).

The differentiation of the groups based on the number of correct answers on subtests 9 and 10 shows the increased burden of working memory in these blocks, such as increasing linguistic (syntactic) complexity by introducing a variety of grammatical constructions into statements. The results showed a reduction in performance (lower scores and longer execution time) proportional to the increase in the extent and syntactic complexity of the stimulus.

One of the most important abilities required to understand longer utterances is working memory, which stores verbal information, allows for comprehension of speech sequences, and organizes responses [8, 9]. According to McNeil and Prescott [22], the RTT provides a direct measure of short-term memory, especially the phonological loop of working memory, comprehension of various types of sentences and their transformations, and understanding of specific vocabulary and certain semantic relations (conditional phrases).

According to the language model proposed by Shalom and Poeppel [44], language processing can be divided into three main processes: analysis, storage, and synthesis, which require the involvement of large brain networks. All three processes appear to be linked to performance on the RTT because the test commands must be analyzed, interpreted based on phonological, syntactic, and semantic processes, and summarized using motor output (which requires visuospatial analysis, planning, coordination, and working memory), perhaps explaining the correlations with cognitive test performance.

A number of studies have consistently shown a decline in working memory with age and how this skill can interfere with the performance of language comprehension tasks [9, 45, 46]. Aging may affect the ability to process large amounts of information, a difficulty that may require additional strategies when applying concurrent tasks, such as listening and manipulating elements [47].

One explanation for the equivalence of the performance of older adults is that they have realized compensation because the working memory (also evaluated in the RTT) is extremely vulnerable to aging. In our study, even the old-old adults showed scores similar to those of adults.

A feature of the RTT is that it allows information to be grouped into meaning units (chunks) for retention and command execution. According to Gilchrist et al. [10], in retention tasks, words tend to be grouped; they are not processed separately, and the same applies to the retaining command of the propositions. In the case of the TT, commands gather propositions whose words are composed in an unpredictable way and therefore do not allow for clustering; there is no syntactic or semantic plausibility. Thus, the words are processed as separate items, which burdens the working memory.

Results reported in the literature regarding the effects of aging on the accuracy of responses on the TT are conflicting. Wertz et al. [21] applied only part 5 of the TT and found a correlation with age and a gradual decline in performance after age of 40. Emery [27] observed a decline in individuals aged 30–93 years, with lower scores in the elderly (75–93 years). By contrast, Peña-Casanova et al. [28] evaluated subjects between 50 and 94 years of age and found little effect of age on TT performance. Snitz et al. [29] assessed elderly over 65 years and showed that performance on the Indiana University version of the TT was associated with younger age. Yang et al. [30] showed that seniors aged 65 years and older performed worse on the TT than did other groups.

The conflicting data can be explained by the different scoring systems used and different age groups studied. With respect to an analysis of execution time on the RTT, the elderly required more time to process information and compensate for possible auditory processing difficulties but exhibited similar results to adults regarding the number of correct answers for the majority of the test.

In this respect, Kim et al. [31] argued that the binary scoring system of hit-error (accuracy) used in scoring the TT is not sensitive enough to detect the effect of aging and established a performance measurement based on response time rather than accuracy. The authors found significant correlations with age above 65 years and noted that time measures can be more sensitive for measuring differences in verbal comprehension in this population.

The facilitation of auditory comprehension, particularly when there is more time to engage the compensation mechanism for signal processing deficit to perform tasks related to this language skill, corroborates data reported in the literature [16].

Similar compensations were observed in studies involving functional magnetic resonance imaging, where performance in sentence comprehension was associated with brain activity in certain areas. Older people, who exhibited similar performance to young people, showed additional activity in those areas where activity is typically found in young people. The elderly activated areas of the right hemisphere related to articulatory recapitulation of the phonological loop [15]. The present study provides evidence for this same phenomenon in the elderly subjects assessed. Therefore, it should be recognized that some aspects of the functioning of working memory resist aging-related loss, such as those related to vocal and subvocal rehearsal, and can support compensation for sentence comprehension.

Similarly, our results show that measures of time, especially execution time, are more sensitive for consistently detecting the effects of aging, regardless of hit analysis (binary or multidimensional), and the differences are evident even in young elderly (from 60 years). The fact that elderly required more time to complete the task but showed similar performance to younger individuals in subtests 1 to 8 of the RTT, which contained commands with simple or coordinated propositions, suggests the integrity of less complex syntactic processes [22].

Aging compensation mechanisms are related to cognitive reserve. Cognitive reserve theory recognizes that factors such as education, parental education, occupation, and reading habits may help maintain the performance of the elderly, particularly, naming, grammar comprehension, and vocabulary tasks [11]. Among these factors, education is highlighted because it is closely related to working memory skills [48] and recruited in the comprehension of syntactically complex sentences. [11]. It is possible that the education factor has provided the elderly maintenance skills necessary for the performance of the RTT, although they needed an increased runtime strategy.

The RTT has been an interesting tool to study the effect of aging on the auditory sentence comprehension. The addition of temporal measures and the correlation with other cognitive tests can refine the reasoning about the underlying processes of syntactic comprehension. This perspective indicates the need for additional studies.

Moreover, the analysis of execution time helps inform the possibilities for cognitive stimulation in the elderly, such as the development of programs related to processing in temporal aspects of working memory.

The main limitation of this study was the absence of reaction time analysis, computerized records, or even videos that allow for qualitative and quantitative refinement of observations such as the nature of test errors. Another important limitation was the absence of functional imaging studies to confirm and describe the nature of the compensation processes.

This study raises the prospect of further investigations, such as studies on the relationship between performance on the RTT and other language parameters, for example, naming, repetition, and written language.

## 5. Conclusions

Young-old and old-old subjects showed similar performance to adults in auditory comprehension task as measured by the number of correct answers on RTT, while differing from adults on the last two subtests. However, elderly required more time to respond to commands. This behavior shows that the execution time measurement is sensitive for detecting the effects of age. Sentence comprehension ability was correlated with performance on cognitive tests, particularly, attention and working memory. Healthy aging is characterized by the ability to compensate for difficulties in linguistic processing, which allows the elderly to maintain normal function in everyday life situations.

## Figures and Tables

**Table 1:** Examples of the complexity of RTT commands in each block.

Block	Examples
1	Touch the black circle
2	Touch the big green circle
3	Touch the green square and the black square
4	Touch the big green square and the little black square
5	Put the black circle above the white square
6	Put the big red square in front of the big white circle
7	Put the black circle to the left of the white square
8	Put the little green circle to the left of the big red square
9	Instead of the green square, touch the black square
10	Touch the big black square unless you have touched the little red circle

**Table 2:** Demographic and cognitive characteristics of the sample.

Variable	Group 1 mean (SD)	Group 2 mean (SD)	Group 3 mean (SD)	*p* value	Multiple comparisons (<0.05)
Age	54.3 (3.3)	64.3 (3.1)	74.3 (5.4)	<0.001^*∗*^	G1 × G2 × G3
Education	10.9 (3.5)	10.8 (3.5)	10.8 (3.8)	0.991	ns
Gender					
M	5	10	7	0.530	ns
F	19	14	17		
MMSE	28 (1.1)	27.5 (1.8)	27.7 (1.7)	0.511	ns
CCQ	0.2 (0.5)	0.1 (0.3)	0.4 (1.0)	0.584	ns
GDS	1.8 (1.4)	1.3 (1.2)	1.2 (1.0)	0.214	ns
SVF	17.4 (5.2)	16.6 (4.7)	17.7 (3.8)	0.691	ns
DS-DO	5.6 (1.3)	5.6 (0.9)	5.8 (0.9)	0.670	ns
DS-RO	3.5 (1.1)	3.5 (0.9)	4.0 (1.4)	0.603	ns
ASHA-FACS	6.9 (0.0)	6.9 (0.0)	6.9 (0.0)	0.252	ns

SD = standard deviation; F = female; M = male; MMSE = Mini-Mental State Examination; CCQ = Cognitive Change Questionnaire; GDS = Geriatric Depression Scale; SVF = semantic verbal fluency; DS-DO = digit span in direct order; DS-RO = digit span in reverse order; ASHA-FACS = Functional Assessment of Communication Skills for Adults.

^*∗*^Statistically significant difference.

**Table 3:** Performance of groups on the RTT as measured by number of correct answers (accuracy).

Subtest	Group 1 mean (SD)	Group 2 mean (SD)	Group 3 mean (SD)	*p* value	Multiple comparisons (<0.05)
1	5.0 (0.0)	4.8 (0.6)	5.0 (0.0)	0.199	ns
2	4.9 (0.2)	4.7 (0.7)	4.8 (0.5)	0.596	ns
3	4.7 (0.6)	4.8 (0.3)	5.0 (0.0)	0.051	ns
4	4.6 (0.9)	4.7 (0.5)	4.6 (0.5)	0.778	ns
5	4.4 (0.9)	4.2 (1.1)	4.2 (1.0)	0.726	ns
6	4.0 (1.1)	3.9 (1.4)	3.8 (1.0)	0.843	ns
7	4.5 (0.7)	4.2 (1.1)	4.5 (0.6)	0.520	ns
8	4.2 (1.3)	4.1 (1.0)	4.1 (1.1)	0.972	ns
9	4.2 (0.5)	3.6 (0.7)	3.3 (0.8)	<0.001^*∗*^	G1 × G2 G1 × G3
10	3.8 (1.0)	3.1 (1.3)	2.8 (1.4)	0.024^*∗*^	G1 × G2 G1 × G3

Total	44.5 (5.0)	42.6 (5.4)	42.4 (4.8)	0.319	ns

SD = standard deviation.

^*∗*^Statistically significant difference.

**Table 4:** Performance of groups on the RTT as measured by execution time.

Subtest	Group 1 mean (SD)	Group 2 mean (SD)	Group 3 mean (SD)	*p* value	Multiple comparisons (<0.05)
1	3.7 (2.1)	6.4 (4.2)	7.5 (4.6)	0.057	ns
2	5.1 (2.2)	7.0 (3.3)	9.2 (6.1)	0.006^*∗*^	G1 × G3 G2 × G3
3	6.4 (3.5)	9.4 (7.5)	11.2 (7.9)	0.047^*∗*^	G1 × G3
4	9.7 (4.9)	11.7 (8.1)	14.1 (6.6)	0.048^*∗*^	G1 × G3
5	23.1 (7.7)	28.5 (10.6)	31.3 (11.1)	0.017^*∗*^	G1 × G2 G1 × G3
6	26.2 (7.0)	30.8 (10.2)	37.5 (13.9)	0.002^*∗*^	G1 × G3 G2 × G3
7	20.3 (7.0)	24.0 (10.3)	26.9 (8.0)	0.034^*∗*^	G1 × G3
8	18.4 (8.0)	33.4 (11.5)	33.7 (9.7)	0.018^*∗*^	G1 × G2 G1 × G3
9	15.6 (8.5)	22.1 (10.0)	24.7 (9.2)	0.003^*∗*^	G1 × G2 G1 × G3
10	18.8 (10.1)	23.2 (8.5)	25.4 (8.4)	0.047^*∗*^	G1 × G3

Total	148.7 (37.4)	190.1 (52.1)	211.9 (55.4)	0.002^*∗*^	G1 × G2 G1 × G3

SD = standard deviation.

^*∗*^Statistically significant difference.

**Table 5:** Correlations between performance on RTT and cognitive test performance.

	Performance on RTT	
	Total correct answers	Total execution time
MMSE	*r* = 0.344 (*p* = 0.003)^*∗*^	*r* = −0.102 (*p* = 0.392)
SVF	*r* = 0.157 (*p* = 0.186)	*r* = −0.114 (*p* = 0.336)
DS-DO	*r* = 0.308 (*p* = 0.008)^*∗*^	*r* = −0.089 (*p* = 0.456)
DS-RO	*r* = 0.400 (*p* = 0.005)^*∗*^	*r* = −0.022 (*p* = 0.853)

MMSE = Mini-Mental State Examination; SVF = semantic verbal fluency; DS-DO = digit span in direct order; DS-RO = digit span in reverse order.

^*∗*^Statistically significant difference.
